# The Principles and Practice of Ophthalmic Medicine and Surgery

**Published:** 1863-12

**Authors:** 


THE PRINCIPLES AND PRACTICE OF OPHTHALMIC MEDICINE AND SURGERY. By T. WHARTON JONES, F.R.S., Prof. of Ophthalmic Medicine and Surgery in University College, London; Ophthalmic Surgeon to the Hospital, etc.; with One Hundred and Seventeen Illustrations. Third and Revised American Edition, with Additions, from the Second London Edition. Philadelphia: BLANCHARD & LEA. 1863.

This is a neatly executed volume of 455 pages octavo, the author of which, is already well known to the profession. It is an excellent practical treatise on the Medical and Surgical Diseases of the Eye; and is well adapted to the wants, both of the student and the practitioner.

For sale by W. B. KEEN & Co., 148 Lake Street, Chicago.

